# Factors Influencing Mental Health Outcomes Amongst Senescent County Residents

**DOI:** 10.3390/ijerph22030451

**Published:** 2025-03-18

**Authors:** Jack Golder, Evan Lagerberg, William Flanagan, Jennifer Blouin, Corey Horn, Sabrina Avanzato, Ryan Scagliarini, Alexis M. Stoner

**Affiliations:** Edward Via College of Osteopathic Medicine, Spartanburg, SC 29303, USA; jgolder@carolinas.vcom.edu (J.G.); elagerberg@carolinas.vcom.edu (E.L.); wflanagan@carolinas.vcom.edu (W.F.); jblouin@carolinas.vcom.edu (J.B.); chorn01@carolinas.vcom.edu (C.H.); savanzato@carolinas.vcom.edu (S.A.); rscagliarini@carolinas.vcom.edu (R.S.)

**Keywords:** aging, mental health, SEM analysis, ecological model of health

## Abstract

Background: Despite increased mental health awareness and expanded healthcare services in recent years, older adults face significant gaps in mental health support. The purpose of this study was to examine factors affecting mental health among elderly residents in Greenville County, South Carolina, using the ecological model of health. Methods: The ecological model of health was used as a conceptual base for a convenience survey of older adults participating in a community involvement program. These results were analyzed using structural equation modeling. Results: The findings revealed that outlooks on aging significantly influence mental health, particularly through personal and relational factors. This study also found that there is a relationship between the concept of healthy aging and overall mental health. Conclusions: This study underscores the need for targeted interventions that enhance social engagement, strengthen community support, and address societal gaps to improve mental health outcomes and create a supportive environment for elderly populations.

## 1. Introduction

Presently, it is estimated that more than 57 million individuals in the United States are living with a diagnosed mental illness, making it a substantial health concern across all age groups [1]. While screening measures for mental health have increased over the past decade due to rising diagnoses, the stigma surrounding treatment remains pervasive, with over 70% of those diagnosed not receiving treatment [2]. This decision to forego treatment is influenced by various intra- and interpersonal factors, including a lack of understanding about mental illness, limited awareness of treatment options, prejudice against mental health issues, and ongoing health trends in the United States [3].

Since the onset of the COVID-19 pandemic, the healthcare system in the United States has shifted its focus toward enhancing mental health awareness and implementing legislative initiatives [4,5]. Consequently, both inpatient and outpatient healthcare services have expanded, improving access to care for individuals with mental health concerns [6]. However, these changes have not been equally inclusive for the elderly population, with insufficient resources dedicated to bridging the gap in healthcare access between younger and older demographics [7]. This is particularly concerning given the rising rates of depression and anxiety among older adults, with depression being the most prevalent psychiatric disorder in later life [8]. The lack of support for the elderly in mental health treatment options can exacerbate concurrent chronic and acute medical issues such as heart failure, hypertension, and acute injuries [8,9,10].

Though variable, it has been found that along with physical and cognitive decline, there is a decline in mental health amongst adults as they age [11,12], further cemented by the results of this study. This decline is leading to a higher rate of depression amongst aging populations [13] as well as a recent rise in suicide rates amongst specifically elderly men [14]. These trends further stress the need for community strategies targeting the health and well-being of the elderly.

Greenville County, South Carolina, with a population of 558,036 in 2023 [15], has nearly 20% of its population classified as elderly, defined as individuals aged 65 and older [16]. Despite being home to South Carolina’s largest private healthcare system, the county exhibits higher-than-national levels of self-harm, mental disorders, and substance use disorders among both men and women [17]. The gap between the availability and accessibility of care, combined with Greenville County’s aging population, positions the region as a key target for public health intervention and implementation strategies.

The ecological model of health (Table 1) is a framework aimed at understanding the factors influencing healthy lifestyle decisions and how sociocultural elements impact an individual’s health. These factors encompass individual, relationship, community, and societal practices [18]. Previous studies have utilized this model to explore factors influencing diabetes education in Malawi [19], resilience among marginalized communities in the United States [18], and the drivers of hazardous alcohol use in Europe [20].

The objective of this study was to identify the factors influencing mental health and well-being among the elderly residents of Greenville County, South Carolina, using the ecological model of health. This research aims to inform community-driven initiatives, programs, and policy changes to improve mental health outcomes for this population.

## 2. Materials and Methods

### 2.1. Survey Design

This cross-sectional study was approved by Edward Via College of Osteopathic Medicine’s Internal Review Board as an IRB-exempt study secondary to the sole use of survey procedures analyzing public behavior. An online self-administered survey was distributed to a population of adults who were 65 years of age and older who are a part of a community engagement program for elderly individuals at a four-year liberal arts college located in Greenville County, South Carolina, USA. This program offers weekly activities for residents aged 65 and older, including painting, music, physical exercise, and reading, fostering community engagement and enrichment. Study participants were recruited in September 2024 using convenience sampling via community outreach. Recruitment occurred via flyers distributed to members of a local community program and via emails to faculty members. All survey questions were close-ended and formatted for forced responses to prevent incomplete responses. Survey question development was adapted through the University of Michigan National Poll on Healthy Aging [21].

The data obtained were anonymous, personally identifiable information was not collected in the survey, and single-blind survey methods were used to countervail any research bias. Qualtrics XM (Seattle, WA, USA), a HIPAA-compliant software package that uses Transport Layer Security (TLS) encryption for all transmitted data [22], was used to collect participant consent and survey data.

The primary outcome for this study was the current mental health trends of elder adults. Questions regarding several mental health variables, including socialization, the use of technology and electronics, travel, perceived health, and community involvement, were included in this study. Questions and response options for these variables are provided in Table S1.

### 2.2. Proposed Model

**Figure 1:**
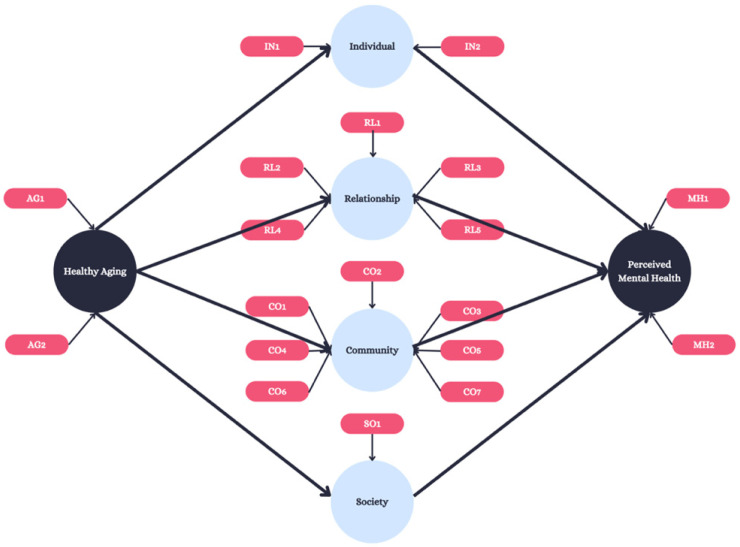
Conceptual model. A map between the theoretical link between aging and mental health outcomes through the use of the ecological model of health.

The ecological model of health (EMH) was used to evaluate the association between residents’ perceived mental health and the factors by which it is influenced, using the mediators of (1) the individual (i.e., feelings of stress or joy); (2) relationships (i.e., the resident’s interactions with those to whom they are closest); (3) community (i.e., the involvement of the resident in attending outings); and (4) society (i.e., the involvement of the resident within their nation and national influences on the resident themselves) (Figure 1).

The model-attributed survey questions and subsequent response options are presented in Table S1. Outlooks on aging were measured via two constructs requiring the individual to associate mental emotions with the future: joy (AG1) and stress (AG2). Perception involved a single 5-point Likert scale of self-reported health, with “excellent = 1”, “very good = 2”, “good = 3”, “fair = 4” and “poor = 5”. The next latent variable, individual, included two questions regarding lack of companionship (IN1) and feelings of isolation (IN2). Relationship had five variables, asking participants to attribute current relationships to joy including those with spouses/partners, children, grandchildren, friends, and neighbors (RL1-RL5). Community was indicated by the out-of-home activities that the participant participated in including their ability to connect with others (CO1-CO3) as well as physically attend events (CO4-CO7). Society was measured through stress attributed to national politics (SO1). Finally, overall mental health was indicated by two variables including the reported current mental health status of the individual, measured on a 5-point Likert scale (“excellent = 1”, “very good = 2”, “good = 3”, “fair = 4” and “poor = 5”) (MH1), and a measurement of the participant’s overall joy (MH2).

### 2.3. Statistical Analysis

**Figure 2:**
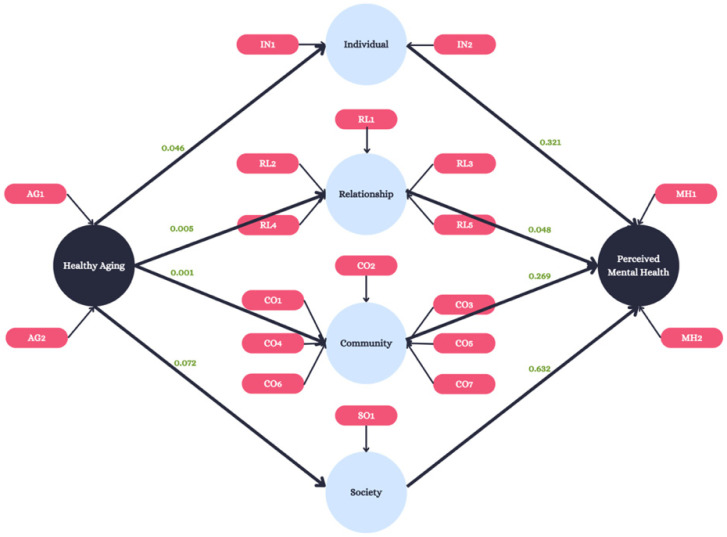
SEM model. The original conceptual model with added *p*-values reported for outer loadings and constructs.

The association between perceived aging and mental health outcomes was tested using structural equation modeling (SEM) [23]. This model has been shown to provide unique insight into the health behavior of an individual due to its ability to conduct multivariate statistical procedures [24] and has been used in previous studies to compare personal attributes to public health characteristics [25]. In addition, this model has been shown to be advantageous for studies with small sample sizes, such as those using nonparametric bootstrapping, further ensuring the correctness and accuracy of the testing [26]. Manifest variables were considered formative when the variable influenced the latent construct, whereas manifest variables were considered reflective if the latent construct influenced the variable [27]. The primary dependent variable, perceived mental health, comprised two reflective manifest variables (MH1-MH2; Figure 1; Table S1). Mediating variables included individual (two manifest variables: IN1-IN2), relationship (five manifest variables: RL1-RL5), community (seven reflective manifest variables: CO1-CO7), and society (one reflective manifest variable: SO1). The primary predictor, outlook on aging, comprised two reflective manifest variables (AG1-AG2; Figure 1). Model fit was evaluated via factor analysis with 1000 iterations and outer loadings for manifest variables meeting or exceeding a threshold of 0.70 [28]. The interpretation of these variables was based on empirical views, with 0.3–0.5 indicating good correlation and anything above 0.70 indicating very good correlation [23]. Construct validity and reliability was tested via the average variance extracted (AVE; threshold = 0.5; Hamid et al., 2014) and the Fornell and Larcker and Heterotrait–Monotrait ratios (both thresholds = 0.85) [29]. Two-tailed, bias-corrected path analysis using nonparametric bootstrapping and 1000 subsamples was used to estimate path coefficients and evaluate model construct relationships using α = 0.05. Smart PLS Software version 3.3.7 (Bönningstedt, Germany) was used for SEM analysis [30] (Figure 2).

## 3. Results

### 3.1. Respondents

In total, 27 completed responses were used for statistical analysis. Median values for model constructs can be found in Table 2. Overall, respondents were found to have a median perceived mental health of 2.0 (“very good”) with a median joy value of 2.0 (“some”). Individual showed a median value of 1.0 (“a lot”) in all categories. Relationship showed a median value of 1.0 (“a lot”) in all categories except for RL5 which had a median value of 2.0 (“some”). Community attributes had mixed median values between 1.0 (“a-lot”) and 2.0 (“some”). Society and aging both contained constructs with median values of 2.0 (“some”).

### 3.2. Model Results

The results of the measurement model factor analysis showed a good model fit (NFI = 0.417; SRMR = 0.147). In addition, construct validity showed an average of 0.63 for all latent variables (Table 2) and most Heterotrait–Monotrait values < 0.85 (Table 3).

Path coefficients were derived using structural equation modeling to test direct and indirect associations between the primary independent variable (outlook on aging) and the primary outcome (perceived mental health). The model indicated that an individual’s relationships affect their perception of mental health (*p* = 0.048; Table 4). There is not sufficient evidence to suggest that individual, community, or societal attributions are associated with mental health outcomes (*p* = 0.321, 0.269, and 0.632, respectively, Table 4). Direct relationships were found between outlooks on aging and individual, relationship, and community attributions (*p* = 0.046, *p* = 0.005, and *p* = 001, respectively, Table 5). Finally, SEM revealed that outlooks on aging are associated with perceived mental health through an overall indirect path (*p* = 0.013) (Table 6).

## 4. Discussion

The primary objective of this study was to determine what intra- and interpersonal factors impacted the mental health outlooks of an aging population. Through the use of the ecological model of health, several significant relationships were identified. First, there is a relationship between aging and individualism, formed relationships, and individuals’ role within their community. Most importantly, it was found that there is an association between mental health and aging in which targeted public health strategies can be implemented.

The results of our study align with the current shift in America’s approach to mental health, particularly for older adults. This is particularly relevant as community programs across the country, much like the community involvement program in our study, aim to enhance mental health for elderly individuals. Our results further emphasize the importance of developing targeted interventions that foster social engagement, strengthen support networks, and address societal challenges that may hinder mental well-being. These findings echo broader efforts to prioritize mental health in aging populations, signaling a critical need for programs and policies that create inclusive and supportive environments for America’s elderly, ultimately improving their overall quality of life [12].

Following the need for increased mental health outcomes amongst elder adults, several programs have been developed to meet this need, targeting socialization, physical activity, hobbies, and other bridging factors [31]. For example, a study in North Carolina concluded that the inclusion of a physical activity program for elder adults improved overall quality of life amongst participating individuals while also allowing for socialization [32]. Similarly, a global systematic review found that social support interventions throughout elder communities resulted in a reduction in diagnoses of depression and other mental health-related disorders [33].

Similarly to the results seen in this study, it has been found that participation in community-based wellness programs and socialization with others greatly influences trends in mental health among elderly populations [33]. This is why the National Council of Aging has a focus on behavioral programs for aging adults, aimed at decreasing the rates of depression/depressive symptoms, substance and/or alcohol abuse, and the implementation of environmental planning focused on creating uplifting environments for this population [34]. Overall, it is programs like these that require further research and implementation, allowing for increased mental health outcomes amongst senescent adults.

A recent study explored the advantages of integrated physical and mental healthcare services for older adults. These services, which included combined physical–mental health wards and multidisciplinary teams, were linked to improvements in care quality, professional satisfaction, and patient engagement. The findings highlight the crucial role of integrated care models in meeting the complex needs of older adults with both physical and mental health challenges [35]. Additionally, a newly proposed framework aims to enhance adult mental health by addressing ageism, fostering age-friendly environments, and promoting seamless care integration across both community and healthcare sectors. This framework offers valuable guidance for shaping policies and practices that support healthy aging and mental well-being [36].

Data collection for our study was confined to residents of Greenville County, resulting in a small sample size for statistical analysis. While we recognize this as a significant limitation, we present statistically significant findings that, despite the sample size, were appropriately tailored for our analysis and can be extrapolated to a larger population. Due to recall bias and the self-report nature of the survey, the validation of survey responses could not be confirmed. While the demographic sample of this survey was designed to mirror that of Greenville County, we recognize that it did not fully capture the county’s diversity. Additionally, the lack of comprehensive demographic data further limits this study’s representation of diverse populations. Despite these limitations, to our knowledge, this is the first analysis of its kind looking into the factors that influence mental health outcomes in elder adults and can be used to further current mental health programs targeting this population.

## 5. Conclusions

In conclusion, this study underscores the critical need for targeted mental health interventions tailored specifically for the elderly population of Greenville County, South Carolina. Despite recent advancements in mental health awareness and care, significant gaps remain, particularly in addressing the unique needs of older adults. Our findings, derived from applying the ecological model of health, highlight that while individual relationships and community involvement play a crucial role in shaping mental health outcomes, societal factors and broader community resources remain inadequately addressed. The results emphasize that improving mental health in this demographic requires a multifaceted approach that includes not only enhancing individual and relational support but also strengthening community and societal frameworks. Initiatives that focus on increasing social engagement, fostering supportive relationships, and developing accessible community-based wellness programs are essential to mitigate the rising rates of depression and anxiety among older adults. For example, creating more community-based programs that encourage interaction, such as senior social clubs, group exercise classes, and volunteer opportunities, can provide older adults with a sense of purpose and connection. Additionally, offering intergenerational programs where younger generations can engage with older adults can foster relationships, reduce feelings of isolation, and improve overall well-being. Moving forward, it is imperative that policymakers and community leaders prioritize these areas, ensuring that mental health interventions are as inclusive and effective as possible, thereby fostering a healthier, more supportive environment for all elderly residents.

## Figures and Tables

**Table 1:** The ecological model of health, adapted from the CDC principles of community engagement.

Model	Definition
Individual	The first level identifies historical biological and personal factors that increase the likelihood of becoming a victim or perpetrator of violence. Some of these factors are age, education, income, substance use, or a history of abuse. Prevention strategies at this level promote attitudes, beliefs, and behaviors that prevent violence. Specific approaches may include conflict resolution and life skills training, social–emotional learning, and safe dating and healthy relationship skills programs.
Relationship	The second level examines close relationships that may increase the risk of experiencing violence as a victim or perpetrator. A person’s closest social circle—peers, partners, and family members—influences their behavior and contributes to their experience. Prevention strategies at this level may include parenting or family-focused prevention programs and mentoring and peer programs designed to strengthen parent–child communication and promote positive peer norms, problem-solving skills, and healthy relationships.
Community	The third level explores the settings, such as schools, workplaces, and neighborhoods, in which social relationships occur and seeks to identify the characteristics of these settings that are associated with becoming victims or perpetrators of violence. Prevention strategies at this level focus on improving the physical and social environment in these settings (e.g., by creating safe places where people live, learn, work, and play) and by addressing other conditions that give rise to violence in communities (e.g., neighborhood poverty, residential segregation, instability, and a high density of alcohol outlets).
Society	The fourth level looks at the broad societal factors that help create a climate in which violence is encouraged or inhibited. These factors include social and cultural norms that support violence as an acceptable way to resolve conflicts. Other large societal factors include the health, economic, educational, and social policies that help to maintain economic or social inequalities between groups in society. Prevention strategies at this level include efforts to promote societal norms that protect against violence as well as efforts to strengthen household financial security, education, and employment opportunities and other policies that affect the structural determinants of health.

**Table 2:** Median values for constructs used throughout the conceptual model.

Construct	Manifest Variable	Median Score (*n =* 27)
Healthy Aging	AG1	2.0
	AG2	2.0
Individual	IN1	1.0
	IN2	1.0
Relationship	RL1	1.0
	RL2	1.0
	RL3	1.0
	RL4	1.0
	RL5	2.0
Community	CO1	2.0
	CO2	1.0
	CO3	2.0
	CO4	2.0
	CO5	1.0
	CO6	1.0
	CO7	2.0
Society	SO1	2.0
Perceived Mental Health	MH1	2.0
	MH2	2.0

**Table 3:** Discriminant validity (Fornell and Larcker) of constructs.

	Community	Relationship	Society
Community	0.647		
Relationship	0.699	0.626	
Society	−0.270	−0.267	1.000

Threshold value = 0.85.

**Table 4:** Discriminant validity (Heterotrait–Monotrait) of constructs.

	Community	Relationship	Society
Community			
Relationship	1.032		
Society	0.392	0.292	

Threshold value = 0.85.

**Table 5:** Structural model results for path coefficient.

Pathway	Beta	T-Statistic	*p*-Value
Aging → Individual	0.555	1.753	0.046
Aging → Relationship	0.357	2.334	0.005
Aging → Community	0.517	3.494	0.001
Aging → Society	−0.341	1.879	0.072
Individual → Perceived Mental Health	0.267	0.891	0.321
Relationship → Perceived Mental Health	0.255	1.546	0.048
Community → Perceived Mental Health	0.401	0.924	0.269
Society → Perceived Mental Health	0.070	0.450	0.632

Beta of 0.30–0.50 = good correlation; >0.70 = very good correlation. Significance = *p* < 0.05.

**Table 6:** Structural model results for indirect effects.

Pathway	Beta	Standard Deviation	T-Statistic	*p*-Value
Aging → Individual Mental Health	0.091	0.129	0.709	0.479
Aging → Relationship → Mental Health	0.207	0.163	1.273	0.203
Aging → Community → Mental Health	0.148	0.173	0.856	0.392
Aging → Society → Mental Health	0.024	0.062	0.385	0.700
Aging → Mental Health	0.423	0.170	2.494	0.013

Beta of 0.30–0.50 = good correlation; >0.70 = very good correlation. Significance = *p* < 0.05.

## Data Availability

The data that support the findings of this study are available from the corresponding author upon reasonable request.

## Supplementary Materials

The following supporting information can be downloaded at https://www.mdpi.com/article/10.3390/ijerph22030451/s1. Table S1. Model constructs, survey items, and response scales for associated constructs.

## References

[1] Mental Health America The State of Mental Health in America. https://mhanational.org/news/mha-releases-2024-state-of-mental-health-in-america-report/.

[2] National Institute of Mental Health (NIMH) Mental Illness. https://www.nimh.nih.gov/health/statistics/mental-illness.

[3] Henderson C., Evans-Lacko S., Thornicroft G. (2013). Mental illness stigma, help seeking, and public health programs. Am. J. Public Health.

[4] Merz S.M., Fache S.M. (2021). Behavioral health in the pandemic: Making the shift from mental illness to mental well-being. Front. Health Serv. Manag..

[5] Xiong J., Lipsitz O., Nasri F., Lui L.M.W., Gill H., Phan L., Chen-Li D., Iacobucci M., Ho R., Majeed A. (2020). Impact of COVID-19 pandemic on mental health in the general population: A systematic review. J. Affect. Disord..

[6] Stepanova E., Thompson A., Yu G., Fu Y. (2024). Changes in mental health services in response to the COVID-19 pandemic in high-income countries: A rapid review. BMC Psychiatry.

[7] Parkar S.R. (2015). Elderly Mental Health: Needs. Mens Sana Monogr..

[8] Bradshaw L.E., Goldberg S.E., Lewis S.A., Whittamore K., Gladman J.R.F., Jones R.G., Harwood R.H. (2013). Six-month outcomes following an emergency hospital admission for older adults with co-morbid mental health problems indicate complexity of care needs. Age Ageing.

[9] Goldberg S.E., Whittamore K.H., Harwood R.H., Bradshaw L.E., Gladman J.R.F., Jones R.G., Medical Crises in Older People Study Group (2011). The prevalence of mental health problems among older adults admitted as an emergency to a general hospital. Age Ageing.

[10] Turana Y., Tengkawan J., Chia Y.C., Shin J., Chen C., Park S., Tsoi K., Buranakitjaroen P., Soenarta A.A., Siddique S. (2020). Mental health problems and hypertension in the elderly: Review from the HOPE Asia Network. J. Clin. Hypertens..

[11] Reynolds C.F., Jeste D.V., Sachdev P.S., Blazer D.G. (2022). Mental health care for older adults: Recent advances and new directions in clinical practice and research. World Psychiatry Off. J. World Psychiatr. Assoc. (WPA).

[12] Ruiz-Comellas A., Valmaña G.S., Peña J.M., Poch P.R., Carrera A.S., Pujol I.C., Baena I.G., Solà À.C., Vila C.S., Gamisans M.F. (2021). Physical activity, emotional state and socialization in the elderly: Study protocol for a clinical multicentre randomized trial. J. Int. Med. Res..

[13] Lee S.H., Lee H., Yu S. (2022). Effectiveness of Social Support for Community-Dwelling Elderly with Depression: A Systematic Review and Meta-Analysis. Healthcare.

[14] Steptoe A., Deaton A., A Stone A. (2015). Subjective wellbeing, health, and ageing. Lancet.

[15] US Census Bureau Census.gov|U.S. Census Bureau Homepage. https://www.census.gov/.

[16] County of Greenville (2022). County of Greenville Budget Document.

[17] Institute for Health Metrics and Evaluation (2016). US County Profile: Greenville County, South Carolina.

[18] Chapter 1: Models and Frameworks|Principles of Community Engagement|ATSDR. http://medbox.iiab.me/modules/en-cdc/www.atsdr.cdc.gov//communityengagement/pce_models.html.

[19] Bamuya C., Correia J.C., Brady E.M., Beran D., Harrington D., Damasceno A., Crampin A.M., Magaia A., Levitt N., Davies M.J. (2021). Use of the socio-ecological model to explore factors that influence the implementation of a diabetes structured education programme (EXTEND project) inLilongwe, Malawi and Maputo, Mozambique: A qualitative study. BMC Public Health.

[20] Tholen R., Wouters E., Ponnet K., De Bruyn S., Van Hal G. (2020). A Social Ecological Approach to Hazardous Alcohol Use among Flemish Higher Education Students. Int. J. Environ. Res. Public Health.

[21] National Poll on Healthy Aging Survey. https://www.healthyagingpoll.org/reports-more/data.

[22] Kang H., Ahn J.-W. (2021). Model Setting and Interpretation of Results in Research Using Structural Equation Modeling: A Checklist with Guiding Questions for Reporting. Asian Nurs. Res. (Korean Soc. Nurs. Sci.).

[23] Rigdon E.E., George (1998). Structural equations modeling. Marcoulides.

[24] Freeze R., Raschke R.L. An Assessment of Formative and Reflective Constructs in IS Research. Proceedings of the ECIS 2007.

[25] Golder J., Jerge M., Sundstrom B., Dziobak M., Hart L.B. (2024). Factors influencing CDC- recommended preventative behaviors through the COVID-19 pandemic in college students. J. Am. Coll. Health.

[26] Dwivedi A.K., Mallawaarachchi I., Alvarado L.A. (2017). Analysis of small sample size studies using nonparametric bootstrap test with pooled resampling method. Stat. Med..

[27] Memon A.H., Rahman I.A. (2014). SEM-PLS analysis of inhibiting factors of cost performance for large construction projects in Malaysia: Perspective of clients and consultants. Sci. World J..

[28] Hair J.F., Hult G.T.M., Ringle C.M., Sarstedt M., Danks N.P., Ray S. (2021). Partial Least Squares Structural Equation Modeling (PLS-SEM) Using R: A Workbook.

[29] Ringle C.M., Wende S., Becker J. (2015). SmartPLS.

[30] Jeste D.V., Depp C.A., Vahia I.V. (2010). Successful cognitive and emotional aging. World Psychiatry Off. J. World Psychiatr. Assoc. (WPA).

[31] Zenebe Y., Akele B., W/Selassie M., Necho M. (2021). Prevalence and determinants of depression among old age: A systematic review and meta-analysis. Ann. Gen. Psychiatry.

[32] El Ibrahimi S., Xiao Y., Bergeron C.D., Beckford N.Y., Virgen E.M., Smith M.L. (2021). Suicide Distribution and Trends Among Male Older Adults in the U.S., 1999–2018. Am. J. Prev. Med..

[33] Chen L., Zhen Z. (2022). Community Participation and Subjective Well-Being of Older Adults: The Roles of Sense of Community and Neuroticism. Int. J. Environ. Res. Public Health.

[34] NCOA, Explore Mental Health Programs for Older Adults. https://www.ncoa.org/article/advancing-behavioral-health-programs-for-older-adults/.

[35] Beishon L., Hickey B., Desai B., Chithiramohan T., Evley R., Subramaniam H., Maniatopoulos G., Rajkumar A.P., Dening T., Mukateova-Ladinska E. (2024). Integrated Physical-Mental Healthcare Services in Specialist Settings to Improve Outcomes for Older People Living With Mental Health Diagnoses: A Systematic Review. Int. J. Geriatr. Psychiatry.

[36] Horgan S., Prorok J., Ellis K., Mullaly L., Cassidy K.-L., Seitz D., Checkland C. (2024). Optimizing Older Adult Mental Health in Support of Healthy Ageing: A Pluralistic Framework to Inform Transformative Change across Community and Healthcare Domains. Int. J. Environ. Res. Public Health.

