# Supplementary material for: Factors Influencing Mental Health Outcomes Amongst Senescent County Residents
Source: Int J Environ Res Public Health. 2025 Mar 18;22(3):451. doi: 10.3390/ijerph22030451 (PMC11942172; doi:10.3390/ijerph22030451)
Supplement: Supplementary file 1 [file ijerph-22-00451-s001.zip › ijerph-3410540-supplementary.pdf]

International Journal of
Environmental Research and Public Health

MDPI

Table S1. Model constructs, survey items, and response scales for associated constructs

Construct	Manifest Variable	Survey Question	Response Options
Healthy Aging	AG1	How much JOY do you feel when thinking about the future?	1= a lot<br>2= some<br>3= very little<br>4= none
	AG2	How much STRESS do you feel when thinking about the future?	1= a lot<br>2= some<br>3= very little<br>4= none
Individual	IN1	In the past year, how often have you felt a lack of companionship?	1= hardly ever<br>2= some of the time<br>3= often
	IN2	In the past year, how often have you felt isolated from others?	1= hardly ever<br>2= some of the time<br>3= often
Relationship	RL1	To what extent have the following relationships been a source of JOY for you? Spouse/Partner	1= a lot<br>2= some<br>3= very little<br>4= none<br>5= not applicable
	RL2	To what extent have the following relationships been a source of JOY for you? Children	1= a lot<br>2= some<br>3= very little<br>4= none<br>5= not applicable
	RL3	To what extent have the following relationships been a source of JOY for you? Grandchildren	1= a lot<br>2= some<br>3= very little<br>4= none<br>5= not applicable
	RL4	To what extent have the following relationships been a source of JOY for you? Friends	1= a lot<br>2= some<br>3= very little<br>4= none<br>5= not applicable
	RL5	To what extent have the following relationships been a source of JOY for you? Neighbors	1= a lot<br>2= some<br>3= very little<br>4= none<br>5= not applicable

Community	CO1	To what extent have the following activities been a source of JOY for you? Connecting with others by phone or virtually	1= a lot<br>2= some<br>3= very little<br>4= none<br>5= not applicable
	CO2	To what extent have the following activities been a source of JOY for you? Connecting with others in person	1= a lot<br>2= some<br>3= very little<br>4= none<br>5= not applicable
	CO3	To what extent have the following activities been a source of JOY for you? New or strengthened friendships or relationships	1= a lot<br>2= some<br>3= very little<br>4= none<br>5= not applicable
	CO4	To what extent have the following activities been a source of JOY for you? Attending events	1= a lot<br>2= some<br>3= very little<br>4= none<br>5= not applicable
	CO5	To what extent have the following activities been a source of JOY for you? Spending time on hobbies, skills, or projects	1= a lot<br>2= some<br>3= very little<br>4= none<br>5= not applicable
	CO6	To what extent have the following activities been a source of JOY for you? Volunteering or helping others	1= a lot<br>2= some<br>3= very little<br>4= none<br>5= not applicable
	CO7	To what extent have the following activities been a source of JOY for you? Attending spiritual or religious activities	1= a lot<br>2= some<br>3= very little<br>4= none<br>5= not applicable
Society	SO1	To what extent have the following caused you STRESS? National events/politics	1= a lot<br>2= some<br>3= very little<br>4= none<br>5= not applicable
Perceived Mental Health	MH1	In general, how would you rate your mental health?	1= Excellent<br>2= Very Good<br>3= Good

			4= Fair<br>5= Poor
	MH2	Generally, how much JOY do you feel these days?	1= a lot<br>2= some<br>3= very little<br>4= none
